# Exhaled Nitric Oxide Is Useful in Symptomatic Radioactive Pneumonia: A Retrospective Study

**DOI:** 10.1155/2017/5840813

**Published:** 2017-09-24

**Authors:** Jiancheng Li, Xiaobin Fu, Jie Fu

**Affiliations:** ^1^Department of Radiation Oncology, Fujian Provincial Tumor Hospital, Provincial Clinical College of Fujian Medical University, Fujian 350014, China; ^2^Provincial Clinical College of Fujian Medical University, Fujian 350014, China; ^3^Department of Radiation Oncology, Shanghai Jiao Tong University Affiliated Sixth People's Hospital, Shanghai 200233, China

## Abstract

The aim was to defect the exhaled nitric oxide (eNO) prediction value of symptomatic radioactive pneumonia (SRP). 64 cases of lung cancer or esophagus cancer, who had the primary radiotherapy (intensity-modulated radiation therapy), were included from 2015 June to 2016 January. During the following, the patients were divided: the symptomatic radiation pneumonia group (SRP, with the CTCAE v4.0 score > 2) and the asymptomatic radiation pneumonia group (ASRP, with CTCAE v4.0 score ≤ 1). All the patients were measured eNO before and at the end of thoracic radiotherapy and gain the posttherapy eNO value and the eNO ratio (posttherapy eNO value/pretherapy eNO value), then the predictive values of eNO toward SRP were measured using the receiver-operating characteristic (ROC). 17 cases were included in the SRP group and the other 47 were included in the ASRP group. The posttherapy eNO was 29.35 (19~60) bbp versus 20.646 (11~37) (*P* < 0.001), and the ratio was 1.669 (0.61~3.5) versus 0.920 (0.35~1.5) (*P* < 0.01) (symptomatic versus asymptomatic). ROC showed that the cutoff value of SRP was 19.5 bbp (posttherapy eNO, area under concentration-time curve (AUC) = 0.879) and 1.305 (eNO ratio, AUC = 0.774), which meant that posttherapy eNO and eNO ratio were useful in finding SRP.

## 1. Introduction

Cancer remains the leading cause of death globally. The International Agency for Research on Cancer (IARC) recently estimated that 7.6 million deaths worldwide were due to cancer with 12.7 million new cases per year being reported worldwide [1]. Radiation is a physical agent, which is used to destroy cancer cells by damaging the genetic material of cells and thus blocking their ability to divide and proliferate further which depends on the high-energy radiation [2], which is one of the main methods of modern tumor therapies, due to the annually increased incidence of thoracic malignant tumors, such as thoracic malignant tumors in lung cancer, breast cancer, esophageal cancer, or thymic cancer which all require chest radiotherapy [3]. While unlike most other applications involving radiation, the intention of radiation therapy is to deliver high doses of radiation to diseased tissue, constrained by the effects of radiation to healthy tissue [4]. Chest radiotherapy can cause different levels of radioactive injury in normal lung tissues adjacent to the tumor, which mainly appears early acute inflammatory radiation pneumonia and later radiation pulmonary fibrosis [5]. Radiation pneumonia (RP) mainly manifests as fever, cough, breathing difficulty, or even respiratory failure in severe cases [6], so it seriously restricts the increase of radiation dose, leads to reduced local control of chest tumors, and even interrupts radiation therapy [7]. With the development of radiotherapy technologies, the incidence of RP has been decreased significantly, but it is still about 25% [8], and no effective prediction method has been developed yet.

Exhaled nitric oxide (eNO) can be used to detect acute and chronic chest inflammation [9, 10]. Compared with other examination methods, it is noninvasive, timesaving, economical, and reliable, so it is widely used to monitor airway inflammation and evaluate the efficacy of hormone therapy [11, 12]. However, the relationship between eNO and RP is rarely mentioned. This study performed prospective research toward 64 lung cancer or esophageal cancer patients who underwent 3D conformal radiotherapy, aiming to explore the predictive values of eNO toward RP.

## 2. Materials and Methods

### 2.1. Inclusion Criteria

(1) Pathologically or cytologically confirmed as lung cancer, esophageal cancer, or thymoma; (2) without contraindications; (3) the Karnofsky score ≥ 70 points; (4) without a history of asthma and chronic obstructive pulmonary disease (COPD); (5) without serious benign lung disease; (6) had not received previous chest radiation treatment; and (7) with an expected postradiotherapy and survive more than 6 months.

### 2.2. General Information

A total of 64 patients with lung cancer or esophageal cancer were admitted from June 2015 to January 2016 for the first-stage radiotherapy, including 22 cases of lung cancer (17 males and 5 females, aging 43–78 years, with the median age as 61 years; 13 cases of squamous cell carcinoma, 7 cases of adenocarcinoma, and 2 cases of small-cell lung cancer; 1 case was in phase I, 17 cases were in phase III, and 3 cases were in phase IV) and 42 cases of esophageal cancer (35 males and 7 females, aging 43–81 years, with the median age as 61 years; all the 42 cases were squamous cell carcinoma, including 2 cases in phase I, 4 cases in phase II, 28 cases in phase III, and 7 cases in phase IV). All the 64 patients agreed to participate in RP, eNO determination, and computer tomography (CT) scan. This study was conducted in accordance with the declaration of Helsinki. This study was conducted with an approval from the Ethics Committee of Shanghai Jiao Tong University Affiliated Sixth People's Hospital. Written informed consent was obtained from all participants.

### 2.3. RP

All the patients were treated with conformal intensity-modulated radiation. Before radiotherapy, the thoracic and abdominal part was fixed using one thermoplastic sheet or vacuum pad for computed tomography- (CT-) simulating positioning scan, with the scan area ranging from the second cervical vertebra to the second lumbar spine (layer spacing as 5 mm). The images were then transmitted to the treatment planning system so as to outline the tumor area and endangered organs. The gross tumor volume (GTV), clinical tumor volume (CTV), and planned tumor volume (PTV) were sketched according to the criteria of tumor sketching issued by the Department of Thoracic Radiotherapy, Fujian Cancer Hospital. Prescription dose: 50–63 Gy, median dose: 60 Gy. Bilung V20 ≤ 25–30%, average bilung dose: 15 Gy, bilung V5 < 65–70%, the heart V40 ≤ 40 Gy, and the maximum dose of the spinal cord < 45 Gy.

### 2.4. Assessment of RP

The clinical symptoms and imaging findings obtained in the postradiotherapy follow-up were evaluated and classified into 5 grades according to common terminology criteria for adverse events, version 4.0 (CTCAE v4.0) [12], namely, grade 0: without clinical symptoms and imaging performance; grade 1 (imaging changes only appear while without clinical symptoms); grade 2 (clinical symptoms appear while not affecting daily life); grade 3 (affecting daily life and require oxygen inhalation); grade 4 (with severe respiratory insufficiency and require continuous oxygen inhalation or assisted ventilation); and grade 5 (death). In this study, the patients were divided: the symptomatic RP group (SRP, with the CTCAE v4.0 score ≥ grade 2) and the asymptomatic RP group (ASRP, with CTCAE v4.0 score as grade 0 or 1) [13].

### 2.5. Follow-Up

All the patients underwent thoracic CT before radiotherapy, as well as 1 month and 4 months after radiotherapy. After radiotherapy, all the patients were regularly followed up the tumor and general conditions, and if severe cough, dyspnea, or other respiratory symptoms occurred, the patient should be required to receive immediate chest and upper abdominal CT scan, and all the chest CT reports were reevaluated by imaging specialists in our hospital.

### 2.6. Measurement of eNO

All the patients were measured eNO before and at the end of thoracic radiotherapy using one Nano Coulomb Nitric Oxide Analyzer (Sunvou, Wuxi, China). All the patients were advised not to smoke 24 hours prior to the eNO measurement so as to avoid the impact of smoking on the measurement results. When measuring, each patient was placed in a comfortable sitting position, wore a disposable sterile mask, and guided 3~5-second deep breathing (close to the total lung capacity) and immediate exhalation. Because eNO is affected largely by the exhaled airflow, so the exhaled airflow rate in the exhalation process needed to reach 5 L/min. ENO was monitored by an internal sensor inside the instrument so as to meet the requirement of exhaled airflow. If one patient's exhaled airflow cannot meet the requirement, one 2 min rest should be required before remeasurement. The data collected at each measurement point were recorded.

### 2.7. Statistical Analysis

The differences in the posttherapy eNO value and the eNO ratio (namely, the posttherapy eNO value/pretherapy eNO value) between groups SRP and ASRP were compared using the Wilcoxon rank sum test, with *P* < 0.05 considered as statistical significance. The predictive values of eNO toward SRP were measured using the receiver-operating characteristic (ROC) curve, and the maximum Jordan index was used to determine the eNO ratio and posttherapy eNO value so as to determine the optimal value for predicting SRP. All the data were calculated using SPSS20.0.

## 3. Results

### 3.1. Changes of eNO

**Figure 1:**
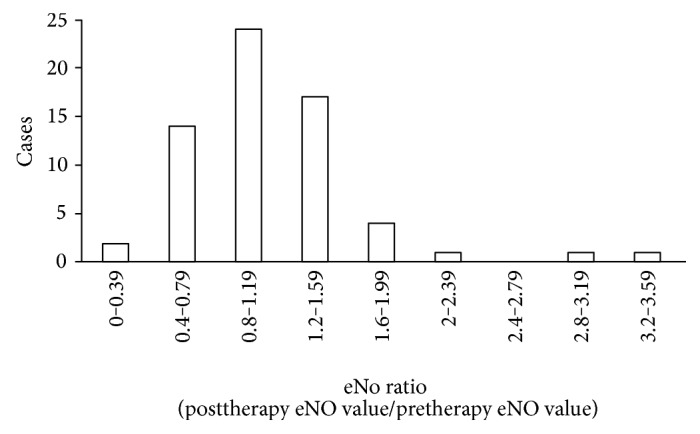
Changes of eNO of all 64 patients.

The eNO values before and after radiotherapy were 23.05 ± 9.59 ppb (amplitude 10~53 ppb) and 22.89 ± 8.60 ppb (amplitude 11~60 ppb). The eNO-changing ratios of all the patients are shown in Figure 1, including 36 patients (56.25%) with the eNO-changing rate ≥ 1 (namely, the posttreatment eNO was increased), 24 patients (37.5%) with the eNO-changing rate ≥ 1.2, 7 patients (7.9%) with the eNO-changing rate ≥ 1.6, and 3 patients (4.68%) with the eNO-changing rate ≥ 2.0.

### 3.2. Evaluation of RP

Among all the 64 patients, 6 patients exhibited both imaging features and severe clinical respiratory symptoms, including fever and cough, which severely affected their daily activities, so they were scored as grade 3 (CTCAE v4.0) and hospitalized for hormone and broad-spectrum antibiotic therapy. 11 patients only exhibited imaging features and mild clinical respiratory symptoms, which did not affect the daily activities, so they were scored as grade 2 (CTCAE v4.0). The 47 patients with only imaging features and no clinical symptom or without any imaging changes nor clinical symptoms were scored as grade 0 or 1 (CTCAE v4.0). The toxicity of RP in all the 64 patients was evaluated (CTCAE v4.0): 47 cases (73.4%) in grades 0-1, 11 cases (17.2%) in grade 2, and 6 cases (9.37%) in grade 3. Group SRP had 17 ≥ grade 2 cases (26.6%) and group ASRP had 47 cases in grades 0-1 (73.4%).

### 3.3. Relationship between eNO and RP

**Figure 2:**
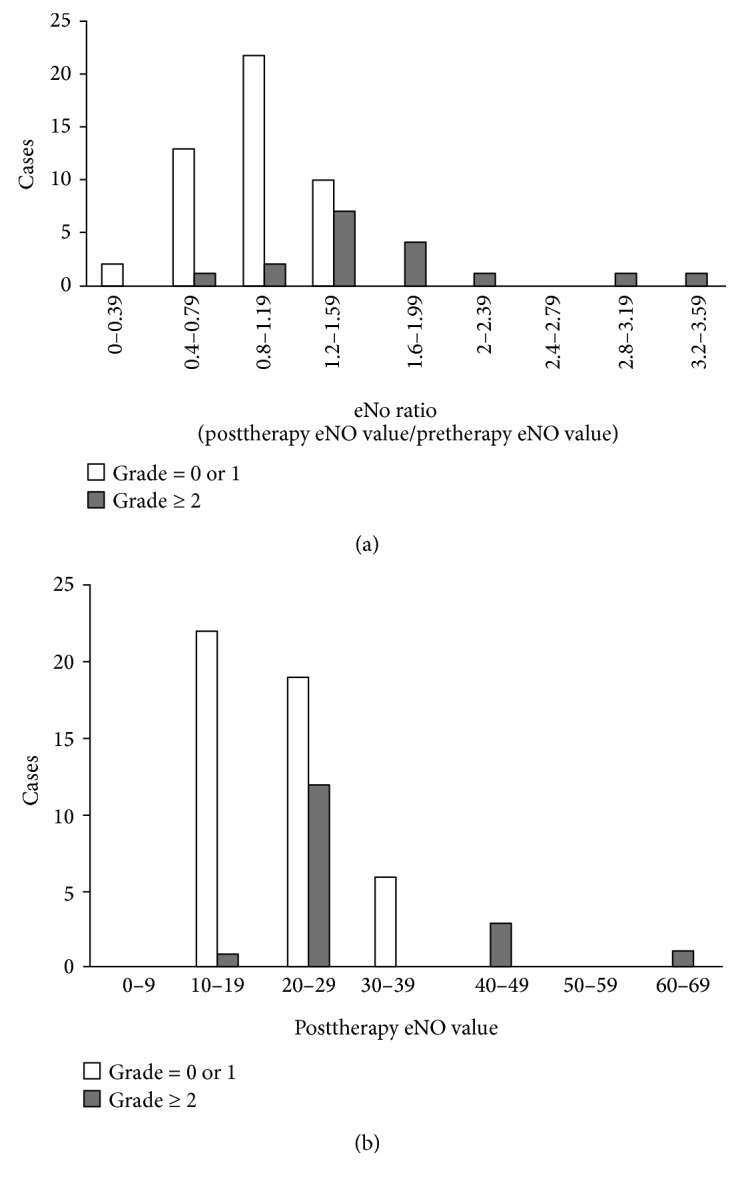
(a) eNO-changing ratios; (b) postradiotherapy eNO values. Orange: group SRP; blue: group ASRP.

The eNO-changing ratios and postradiotherapy eNO values in the two groups are shown in Figure 2.

The comparison of the eNO-changing ratios and the postradiotherapy eNO values revealed that the eNO-changing ratios and the general distribution of the postradiotherapy eNO values were different between the two groups. Group SRP exhibited higher eNO-changing ratio and postradiotherapy eNO value.  Table 1 summarizes the differences in the eNO-changing ratio and the postradiotherapy eNO value in groups SRP and ASRP, and the differences were statistically significant (*P* < 0.05).

### 3.4. Ability of eNO Value in Predicting SRP

The area under concentration-time curve (AUC) of the eNO-changing ratio was 0.879 (95% CI 0.774–0.984). According to the criteria of predictive ability, this indicator exhibited a better predictive ability toward SRP. The Jorden index (sensitivity + specificity −1) revealed that the optimal cutoff value was 1.305, indicating that when the eNO-changing ratio in patients is greater than 1.305, such patient will have a higher chance of SRP.

The AUC of eNO at the end of radiotherapy was 0.774 (95% CI 0.656–0.892). According to the criteria of predictive ability, this indicator exhibited an acceptable predictive ability toward SRP. The Jordan index revealed that the best cutoff was 19.5 ppb, indicating that the patient with the eNO value > 19.5 ppb at the end of radiotherapy will have a higher chance of developing into SRP (Table 2).

## 4. Discussion

In 1980, Furchgott and his colleagues found that endothelial cells can release a diffusible substance that has the effect of relaxing vascular smooth muscle cells and named this diffusible substance as endothelium-derived relaxing factor (EDRF), which was nitric oxide (NO) [14]. In the physiological state, NO can maintain the vascular tension, promote cell growth, or dissolve thrombus, while in the pathological state, NO plays an important role in the processes of a variety of diseases, especially for in the regulation of inflammatory response [15]. NO also contributes to the prevention and treatment of tumors in the radiotherapy process by increasing the cell nucleic acid injury and interrupt intracellular signals [16]. For the meantime, eNO is widely used to detect airway inflammation in patients with asthma and COPD, predict chronic sinusitis or essential hypertension, and diagnose pulmonary embolism [17, 18]. Currently, the relationship between eNO and RP is rarely mentioned. In this study, we used eNO as a biomarker to diagnose RP, measure the eNO value before and after thoracic radiotherapy, and monitor the changes of eNO, thus investigating the predictive value of eNO in RP.

In this study, the incidence of SRP was 26.6%. McCurdy et al. [19] retrospectively analyzed 139 patients who underwent thoracic radiotherapy and found that the incidence of SRP was 58%. However, with wide applications of advanced radiotherapy techniques such as intensity-modulated radiotherapy and proton therapy, the incidence of RP decreases rapidly. Rodrigues et al.'s review of RP [20] pointed out that the incidence of RP fluctuated between 13 and 37%. Carver et al. [21] also reported a lower incidence of RP in his review about the prognosis of patients with lung cancer, ranging from 5% to 15%. In a recent retrospective study which reported 249 patients with lung cancer undergoing cisplatin + etoposide or carboplatin + paclitaxel combined with chest radiotherapy, the incidence of SRP was 29.8%. However, the risk in the patients > 65 years of age, receiving concurrent chemotherapy, and having pneumonia was significantly increased, and their incidence of RP was as high as 50% [22].

Many studies have found that inflammatory biomarkers and doses in blood samples can predict the incidence of RP. Kim et al. [23] reported that the plasma tumor growth factor *β*1 (TGF-*β*1) can be used as a biomarker to predict SRP. That study detected the contents of such plasma cytokines as IL-1, IL-6, IL-10, tumor necrosis factor *α* (TNF-α), and TGF-*β*1 in 34 lung cancer patients at different time points and found the incidence of SRP as 23.5%. The results showed that TGF-*β*1 began to rise after radiotherapy and increased significantly 4 weeks after radiotherapy. TGF-*β*1 was 1.9 ± 0.6 pg/ml before radiotherapy and significantly increased to 3.3 ± 1.7 *μ*g/ml 4 weeks after radiotherapy (*P* = 0.007). There also existed significant association between the TGF-*β*1 level change and the occurrence of RP during radiotherapy. TGF-*β*1 can be used as a risk predictor to predict the occurrence of RP. However, other studies also showed opposite results. Rübe et al. [24] found that TGF-*β*1 cannot be used to predict SRP. The ability of plasma cytokines in predicting RP still remains controversial.

Many studies focused on investigating the relationship between dose factors (mean lung dose MLD, V5, V10, V15, V20, or predictive RP symptoms) and SRP. Nomura et al. [25] retrospectively studied 125 patients with esophageal cancer who underwent concurrent radiotherapy and found that the incidence of SRP was 20.8%. Univariate analysis revealed that clinical stage IV, tumor length, weight loss, and all dosiological factors (such as MLD, V20, V15, V10, or V5) were important factors for the development of SRP. Multivariate analysis revealed that clinical stage IV and all dosiological factors were independent factors for the development of RP. A large number of studies have found that only V20 can predict the occurrence of RP and is significantly related to the incidence and grading of RP. Wang et al. [26] retrospectively analyzed 223 patients with non-small-cell lung cancer who underwent thoracic radiotherapy and found that V5 was the evaluation index with the most significance toward RP. When V5 < 42%, the probability of RP with grade 3 and above was 3%, and when V5 > 42%, the probability increased to 38%; the differences were statistically significant. However, Kristensen et al. [27] indicated that only V10 can be used as a predictor to predict SRP. Currently, there is no consensus about the values of blood indexes and dose factors in predicting SRP.

Our results suggested that the SRP patients had a higher eNO-changing ratio and a higher posttherapy eNO value. McCurdy et al. [28] studied the ability of eNO to predict SRP in 60 patients with esophageal cancer or lung cancer. The results were similar to this study. The eNO-changing ratio can be used to predict the occurrence of SRP after thoracic radiotherapy, which is similar to Guerrero et al. [29]. Guerrero et al. studied 34 patients with esophageal cancer and found that the eNO-changing ratio can be used as a classifier to predict SRP with a smaller average error rate (only 8%). This study found that the best cutoff value of the eNO-changing ratio in predicting SRP was 1.305, and McCurdy et al. found that the patients with the eNO-changing ratio as 1.4 had a higher chance of developing into SRP. Our study also found that the best cutoff value of the posttherapy eNO value for predicting SRP was 19.5 ppb.

ENO has been shown as a good predictive ability toward airway inflammation, chronic obstructive pulmonary disease, and severe asthma and has become a routine clinical examination for such patients. Measuring eNO has such advantages as noninvasive, fast, convenient, and reliable. The measuring instrument used in this study is simple to operate, the operators do not need special technical training, and patients can easily tolerate and be inspected many times. Compared with other studies, we expanded the number of the patients with esophageal cancer and lung cancer, but the number of the patients with SRP was still relatively limited. At the same time, the time points of measuring eNO in this study were less. In order to solve the above problems, we will recruit more patients in future studies so as to fully assess the testing efficacy; furthermore, the patients were instructed to measure the eNO value every other week after radiation therapy so as to explore the changing rules of eNO in radiation therapy. During the study, some potential factors may affect the eNO value. Leon de la Barra et al. [22] reported that the eNO value was significantly different between males and females and the eNO value in female was about 25% less than males. At the same time, smoking also has greater impact in measuring eNO; for example, eNO will be reduced by 50% after smoking, but this impact occurs mainly within 24 hours before the eNO measurement. People with susceptible constitution may exhibit 60% higher eNO than normal people. Virus infection can also easily increase the eNO value; human rhinovirus (HRV) infection can increase the eNO value than normal people, which may be related to that virus infection increases NO so as to eliminate the virus in vivo [23]. Patients with HRV or other viruses may also show such respiratory symptoms as cough or sputum, so it may impact such patients' CTCAE score. At the same time, many drugs can also affect the determination of eNO; certain studies have found that inhaling or orally administrating cortisol hormones can reduce eNO while L-arginine can increase the level of eNO.

Limitation: since patients with atopic dermatitis have higher eNO levels [30], patients with atopic dermatitis were not excluded from the study is a limitation which may bring bias of the study.

In summary, the occurrence of RP is the result of various factors, so the postradiotherapy eNO value and the eNO-changing ratio are useful in SRP. Patients with the postradiotherapy eNO value greater than 19.5 ppb or the eNO -changing ratio greater than 1.305 may have higher risk of SRP, so that such patients should be intervened and treated in advance so as to control and reduce the occurrence of SRP.

## Figures and Tables

**Table 1:** Comparison of eNO-changing ratio and postradiotherapy eNO value between the two groups.

	SRP (*n* = 17)	ASRP (*n* = 47)	*P*
eNO-changing ratio	1.669 (0.61–3.50)	0.902 (0.35–1.5)	*P* = 0.000 < 0.05
Postradiotherapy eNO value (bbp)	29.235 (19–60)	20.646 (11–37)	*P* = 0.001 < 0.05

The data were average values (amplitudes in brackets); the *P* value was calculated using the rank sum test, with *P* < 0.05 considered as statistical significance.

**Table 2:** Evaluation of the ability of NO in predicting SRP.

	AUC	AUC 95% CI		*P*	Jordan index	Sensitivity	Specificity	Best cutoff
eNO-changing ratio	0.879	0.774–0.984		*P* = 0.000 < 0.05	0.72	0.824	0.896	1.305
Postradiotherapy eNO value	0.774	0.656–0.892		*P* = 0.001 < 0.05	0.42	0.941	0.521	19.50
